# Sympathetic Encouragement to Group Clinics

**Published:** 1921-07-09

**Authors:** 


### THE PRIVATE CLINIC SYSTEM IN GREAT BRITAIN.

### Sympathetic Encouragement to Group Clinics.

The resumed discussion on "The Problem of the Private Clinic System in Great Britain" was held at the Royal Society of Medicine on Monday, July 4. The original discussion, it will be remembered, was opened by Sir Thomas Horder on June 16 (see *The Hospital*, June 25), who was followed by several speakers, but the subject was not thrown open for general discussion and the adjourned meeting was to give this opportunity, and, if thought desirable, to move the following resolution:

"That in the opinion of this meeting the time is ripe for the formation of Group Clinics in this country, and that sympathetic encouragement should be given to them by the profession and by its governing bodies."

The first speaker was Dr. Lapthorn Smith, who described the arrangements at the Mayo Clinic, Rochester, those of another private hospital under the group system at Battle Creek, Michigan, and a third at Baltimore, whose chief is a director of the Johns Hopkins Hospital in that city. He urged the economy in money and in time of the private clinic system, both to patients and doctors, and stated that the profits for one year of the private clinic at Battle Creek, which contains now 1,000 patients and has a staff of 1,000, were £12,000, while at a clinic in Vienna of 300 to 500 beds 10 per cent. has been paid ever since it was started. He protested against ornamental buildings in fashionable localities and the sinking of a large amount of capital to ensure the buildings lasting for long periods, say a hundred years. Neither of these methods is necessary, as is shown by the building at Rochester, which is a plain square wooden one of simple plan. Here there is a graded scheme of payment, the rich paying for the poor. Dr. Lapthorn Smith's suggestion for a private clinic for London is that a surgical baronet and a medical knight who have left their hospital should form a team and engage a few young men to assist them. They should form a company with a few thousand shares, themselves and other members of the profession taking them up, which he believes would pay them as well or better than some other concerns in which medical men invest their money. Dr. C. O. Hawthorne followed with a vigorous objection to committing himself without more detailed information to the approval of any of the schemes already submitted, which differ widely from one another. He took it that the common aim and purpose is the establishment of a system or combination variously termed private clinic, group medicine, team work; but these terms, though treated as practically equivalent, received from different speakers widely different and often mutually inconsistent interpretations. To vote on the resolution before the meeting would be to vote for a phrase. Which group clinic was referred to: that of Mr. Dickie, with its expensive buildings, or the scheme now under the control and direction of Dr. Hurst, or that under a group of practitioners as sketched by Sir T. Horder? He urged that the spirit and aim of medical practice are entirely different from the spirit and aim of medical research, and to argue that methods which are necessary or applicable in one case are necessary or applicable in the other is to indulge in a gross and palpable fallacy. He holds there is no objection to combination in medical practice, provided only that there is secured first of all as a commanding influence the welfare of the patient, and secondly, that the authority and responsibility of individual medical practitioners are not obscured behind a crowd of counsellors and are not covered by an enormous institute or organisation. Dr. Hawthorne dealt critically with the various schemes which had been presented. If, as had been stated, medical knowledge is so extensive and detailed that no single practitioner can compass it, it behoves that practitioner not to entrench himself behind these limitations, but to get as near as possible to the ideal—this is, that training both before and after graduation shall be directed to the acquisition of the widest possible knowledge and skill. The group system, he avers, has a tendency in exactly the opposite direction. The hospital patient is not the patient of an institution, or of an organisation, or of a group; he is the patient of an individual responsible medical practitioner, and that practitioner is and must be a free man.

Mr. A. W. Shearn, Dr. Charles F. Harford, Dr. Stanley George, Dr. Chas. Gray, and Dr. Carnegie Dickson took part in the discussion which followed, in which all showed themselves to be in favour of some private clinic system. Mr. Shearn suggested that a cast-iron plan is undesirable; what might do for one part of the country might prove unsuitable for another, and experiments are needed so that experience may be gained. Reform in the present conditions must come, and it were better it should come from within than without. Sir T. Horder, in summing up, said that Dr. Hawthorne had complained of the lack of preciseness in the details of any scheme put forward, but it was not intended to be precise in respect of detail, and he questioned whether preciseness in this regard would have produced a more favourable reception. He believes that the modern tendency to combine rather than to divorce practice from research is a good one, and the experience of the Rockefeller Institute is that if research is to be helpful it must be combined with practice. He admitted that when he was in the United States what he saw of group work damped rather than warmed his ardour as to clinics, which are so big there that they cannot be run with that philanthropic spirit and freedom from financial interest which English people have been assured of. He urged that current hospital practice is not the last word in medicine and surgery. Group medicine will be the next development, and therefore it should receive encouragement. The resolution already quoted was then moved by Sir Thomas Horder, and seconded by Dr. Drury Pennington, Dr. Hawthorne proposing the following amendment: "That until a precise definition and details are submitted this meeting prefers to abstain from pronouncing an opinion on any suggested modification of the existing methods of medical practice." This was seconded by Dr. Fincucane and supported by Dr. Harvey Hilliard, who suggested that if the resolution were passed it would go out as voicing the opinion of the Royal Society of Medicine, and its effect would be to strengthen the hands of the Government to promote a system of clinics, in the first place for the use of insured persons, and ultimately to extend them. If the Ministry of Health did not start these private clinics, who else was going to? How could the finance be provided? The Chairman said the resolution would not commit the Royal Society of Medicine; it was merely an attempt to get at the opinion of the meeting. He then put the amendment, which was declared lost, only seven voting for it. The resolution was put, sixteen voting in favour and six against, and it was carried. The Chairman remarked that example is better than precept, and it now remains with those who are keen on the matter to take counsel together, and with their money and brains start an experimental scheme.

